# Comparative transcriptomics revealed neurodevelopmental impairments and ferroptosis induced by extremely small iron oxide nanoparticles

**DOI:** 10.3389/fgene.2024.1402771

**Published:** 2024-05-17

**Authors:** Zhaojie Lyu, Yao Kou, Yao Fu, Yuxuan Xie, Bo Yang, Hongjie Zhu, Jing Tian

**Affiliations:** ^1^ Key Laboratory of Resource Biology and Biotechnology in Western China, Ministry of Education, College of Life Sciences, Northwest University, Xi’an, China; ^2^ Center for Automated and Innovative Drug Discovery, School of Medicine, Northwest University, Xi’an, China

**Keywords:** high-throughput sequencing, RNA-seq, WGCNA, nervous system, neurotoxicity, ferroptosis, ESIONPs

## Abstract

Iron oxide nanoparticles are a type of nanomaterial composed of iron oxide (Fe_3_O_4_ or Fe_2_O_3_) and have a wide range of applications in magnetic resonance imaging. Compared to iron oxide nanoparticles, extremely small iron oxide nanoparticles (ESIONPs) (∼3 nm in diameter) can improve the imaging performance due to a smaller size. However, there are currently no reports on the potential toxic effects of ESIONPs on the human body. In this study, we applied ESIONPs to a zebrafish model and performed weighted gene co-expression network analysis (WGCNA) on differentially expressed genes (DEGs) in zebrafish embryos of 48 hpf, 72 hpf, 96 hpf, and 120 hpf using RNA-seq technology. The key hub genes related to neurotoxicity and ferroptosis were identified, and further experiments also demonstrated that ESIONPs impaired the neuronal and muscle development of zebrafish, and induced ferroptosis, leading to oxidative stress, cell apoptosis, and inflammatory response. Here, for the first time, we analyzed the potential toxic effects of ESIONPs through WGCNA. Our studies indicate that ESIONPs might have neurotoxicity and could induce ferroptosis, while abnormal accumulation of iron ions might increase the risk of early degenerative neurological diseases.

## Introduction

Iron oxide nanoparticles, including magnetite (Fe_3_O_4_), hematite (α-Fe_2_O_3_), and maghemite (γ-Fe_2_O_3_) NPs as well as modified products, have been widely used in drug carriers and imaging (Lee et al., 2015). For better absorption and imaging, extremely small iron oxide nanoparticles (<5 nm in diameter) (ESIONPs) have been synthesized and modified. ESIONPs have shown the great application values in magnetic resonance imaging (MRI) due to their unique properties, such as switchable contrast signals and high biocompatibility (Kim et al., 2011; Shen et al., 2017; Cao et al., 2020). In addition, ESIONPs are also used as highly sensitive probes for detecting tumors and other lesions (Groult et al., 2021; Mishra et al., 2022; Zhang et al., 2022). As a component of nanotechnology, the application of ESIONPs in different fields is rapidly increasing, and understanding their potential cytotoxicity and mechanism is crucial for the safety of their application (Mohammadinejad et al., 2019).

Related studies have shown that upon ingestion, iron oxide nanoparticles can impact various organs and tissues in the human body. For example, Fe_3_O_4_ nanoparticles reduced neuronal activity, triggered oxidative stress, and might be related to the development of neurodegenerative diseases (Wu et al., 2013); ultra-small superparamagnetic iron oxide NPs accumulated in the lower digestive tract and induced cellular autophagy (Schütz et al., 2014). In addition, the spleen is the main organ responsible for clearing iron oxide nanoparticles from the systemic circulation. The proteomic analysis results showed that iron oxide nanoparticles could promote autophagy and lysosomal activation of splenic macrophages through the AKT/mTOR/TFEB signaling pathway (Han et al., 2022). Moreover, iron oxide nanoparticles detected in the environment also increased the risk of early neurodegenerative diseases among urban residents (Calderón-Garcidueñas et al., 2022). Compared with iron oxide nanoparticles, the potential toxic effects of ESIONPs with higher adsorption capacity and accumulation on the human body still need to be systematically investigated and summarized (Kim et al., 2011).

Zebrafish has a high degree of genetic homology with humans and is widely used to detect the toxicity of nanomaterials (Yang et al., 2018; Qiu et al., 2019). Previous studies showed that iron oxide nanoparticles could penetrate the chorion and act directly on the zebrafish embryos, leading to death, malformation, developmental delay, hatching failure, oxidative stress, and the alteration of redox homeostasis (Pitt et al., 2018; Chemello et al., 2019; Pereira et al., 2020; Thirumurthi et al., 2022). The accumulation of iron oxide nanoparticles in zebrafish larvae also caused the obvious cardiotoxicity, characterized by slowed heart rate, pericardial edema, and cardiac hemorrhage (Pereira et al., 2020). Therefore, zebrafish is suitable as a model animal to detect the toxicity of ESIONPs.

In this study, for the first time, we dynamically analyzed the toxicity of ESIONPs (∼3 nm in diameter) at multiple embryonic development stages by weighted gene co-expression network analysis (WGCNA). The purpose is to explore the impact of ESIONPs on gene expression at different stages of embryonic development, identify the central regulatory genes and related mechanisms affected. It will help evaluate the safety of ESIONPs application, as well as provide valuable insights for the research of other NPs.

## Materials and methods

### Zebrafish husbandry and embryo collection

Zebrafish (*Danio rerio*) was raised according to standard protocols. The following zebrafish lines were used: AB wild-type (wt) strain, and transgenic *Tg(eef1a1l1:EGFP)* expressing enhanced green fluorescent protein (GFP) in neuron cells. Zebrafish embryos were obtained by natural spawning, collected within 30 min after fertilization, and cultured at 28.5°C (Tian et al., 2019). To evaluate the toxicity of ESIONPs, a dose–response analysis was carried out to determine the median lethal dose (LC50). The zebrafish embryos at 4 h post-fertilization (hpf) were distributed in 6-well plates (30 for each group), and exposed to ESIONPs suspensions at different concentrations (0 mg/L, 10 mg/L, 20 mg/L, 30 mg/L, 40 mg/L, 60 mg/L, 80 mg/L, and 100 mg/L). The medium was changed every 24 h. The survival rate was determined every day by counting the embryos that survived. The exposed embryos were collected at indicated stages for different analysis. Embryos from each group were observed and photographed taken an SMZ25 stereomicroscope with a DS-Ri2 digital camera (Nikon, Japan). All experimental procedures on zebrafish were approved by the Institutional Animal Care and Use Committee of Northwest University and carried out in accordance with the approved guidelines (NWU-AWC-20190103Z).

### RNA library preparation and sequencing

Embryos in 40 mg/L ESIONP-exposed group and control group at 48 hpf, 72 hpf, 96hpf, and 120 hpf were collected and used for total RNA extraction. mRNA was isolated using the NEBNext PolyA mRNA Magnetic Isolation Module (New England Biolabs, Ipswich, MA, United States). Libraries were prepared with the NEB Next Ultra Directional RNA Library Prep Kit (New England Biolabs, United States), and subjected to Illumina sequencing with paired end 2 × 150 as the sequencing mode. The clean reads were mapped to reference genome (*D. rerio*: NCBI_GRCz11). Gene expression levels were estimated using FPKM (fragments per kilobase of exon per million fragments mapped) by StringTie v1.3.4d (Pertea et al., 2015). Differential expressed genes (DEG) were measured using R package, edgeR v3.24.2 (Robinson et al., 2010). The false discovery rate (FDR) was used to calculate the adjusted *p*-value in multiple testing in order to evaluate the significance of the differences. Here, only gene with an adjusted q-value < 0.05 and |log2FC| ≥ 1 were used for subsequent analysis. The raw sequence data reported in this paper have been deposited in the Genome Sequence Archive (Chen et al., 2021) in National Genomics Data Center (CNCB-NGDC Members and Partners, 2024), China National Center for Bioinformation/Beijing Institute of Genomics, Chinese Academy of Sciences (GSA: CRA016266) that are publicly accessible at https://ngdc.cncb.ac.cn/gsa.

### Weighted gene co-expression network analysis (WGCNA)

A weighted gene co-expression network analysis was performed using the WCGNA package in R (Langfelder and Horvath, 2008). Samples were clustered by *hclust* to filter outliers (h > 15). In order to construct scale-free network, the optimal soft-thresholding power β was defined by picking Soft Threshold function (β = 6, *R* ^2^ ≥ 0.8). Based on pairwise correlations between genes, genes with similar expression patterns were clustered into a group through a TOM clustering tree according to the dynamic tree cut method, and similar groups were combined into one module. The key hub genes, which were the node of co-expression network, were defined based on the connectivity by the CytoHubba plugin in Cytoscape v3.9.1.

### Enrichment analysis

Gene ontology (GO) terms and Kyoto Encyclopedia of Genes and Genomes (KEGG) pathways were carried on DEGs (Gene Ontology Consortium, 2021; Kanehisa et al., 2023). Enrichment analysis was performed using the R package “clusterProfiler” (Wu et al., 2021). GO terms and KEGG analysis with corrected *p*-value < 0.05 were considered to be significantly enriched (Wang et al., 2021).

### Real-time quantitative PCR (qRT-PCR) analysis

Total RNA was isolated from zebrafish embryos in each group using TRIzol™ reagent (Ambion, United States). The cDNA was synthesized using the SuperScriptIII (Invitrogen, United States), according to the manufacturer’s protocol. qPCR was conducted using SYBR FAST Universal qPCR kit (KAPA, Germany) and ViiA 7 Real-Time PCR System (ABI, United States), as described previously (Wang et al., 2021). Primer sequences were shown in Supplementary Table S1.

### Analysis of skeletal muscle structure by birefringence

Zebrafish embryos at 96 hpf were anesthetized with tricaine (0.04%) and embedded in 5% methylcellulose to score the skeletal muscle lesions. Birefringence was imaged under SMZ25 stereo microscope equipped with a DS-Ri2 digital camera (Nikon, Japan), as previously described (Lu et al., 2021). For quantification analysis, 10 somites between the levels of somite 5 to 15 were imaged per embryo.

### Tracking of swimming behavior

For locomotion tracking, single zebrafish larvae (treated with or without ESIONP) developed to 120 hpf was placed in individual wells of 24-well cell culture plate containing approximately 500 µL embryo medium. Swimming behavior was monitored at room temperature using a DanioVision system and EthoVision XT 11.5 locomotion tracking software (Noldus Information, Netherlands), according previously described (Lu et al., 2021).

### Oxidative stress detection

The oxidative stress and damage caused by ESIONP was detected by measuring ROS production in zebrafish embryo (Zhu et al., 2022). Briefly, embryos at 72 hpf treated with or without ESIONPs were stained with an oxidation-sensitive fluorescent probe dye, dichloro-dihydro-fluorescein diacetate (DCFH-DA) (Beyotime, China) at a final concentration of 20 μg/mL. Stained embryos were incubated at 28°C for 1 h and then washed with PBS. The photos were taken under a fluorescence microscope with a DS-Ri2 digital camera (Nikon, Japan). The fluorescence intensity of embryos was quantified using ImageJ software (NIH, United States).

### Apoptosis analysis

To detect apoptotic cells in zebrafish embryos, acridine orange (AO), a fluorescent dye was used. Zebrafish larvae developed to 96 hpf were incubated with 10 μg/mL AO staining solution (Beyotime, China) at 28.5°C for 30 min in the dark, and rinsed with PBS (Zhu et al., 2022). The zebrafish embryos were observed and recorded under a fluorescence microscope with a DS-Ri2 digital camera (Nikon, Japan). The intensity of the fluorescence signal was measured and analyzed using ImageJ software (NIH, United States).

### Statistical analysis

Each experiment was repeated at least three times. All data were presented as the mean ± SD. Student’s *t*-test was applied for comparisons among different groups. *p-*value < 0.05 was considered significant.

## Result

### Construction of the stage-specific gene co-expression networks via WGCNA

To assess the toxicity of ESIONPs, zebrafish embryos were exposed to different concentrations of ESIONPs (0, 10, 20, 30, 40, 60, 80, and 100 mg/L), the survival rate was counted at 24, 48, 72, 96, and 120 hpf (Supplementary Figure S1A). The LC50 of ESIONPs was determined at 72 hpf (Supplementary Figure S1B). The degrees of malformations are defined in 4 classes, including dorsal bending, shortened body length, yolk sac swelling, cardiac edema, smaller eyes, and head (Supplementary Figure S1C). A concentration of 40 mg/L was chosen for subsequent experiments, to ensure the maximum survival rate of embryos while including diverse morphological abnormalities.

**FIGURE 1:**
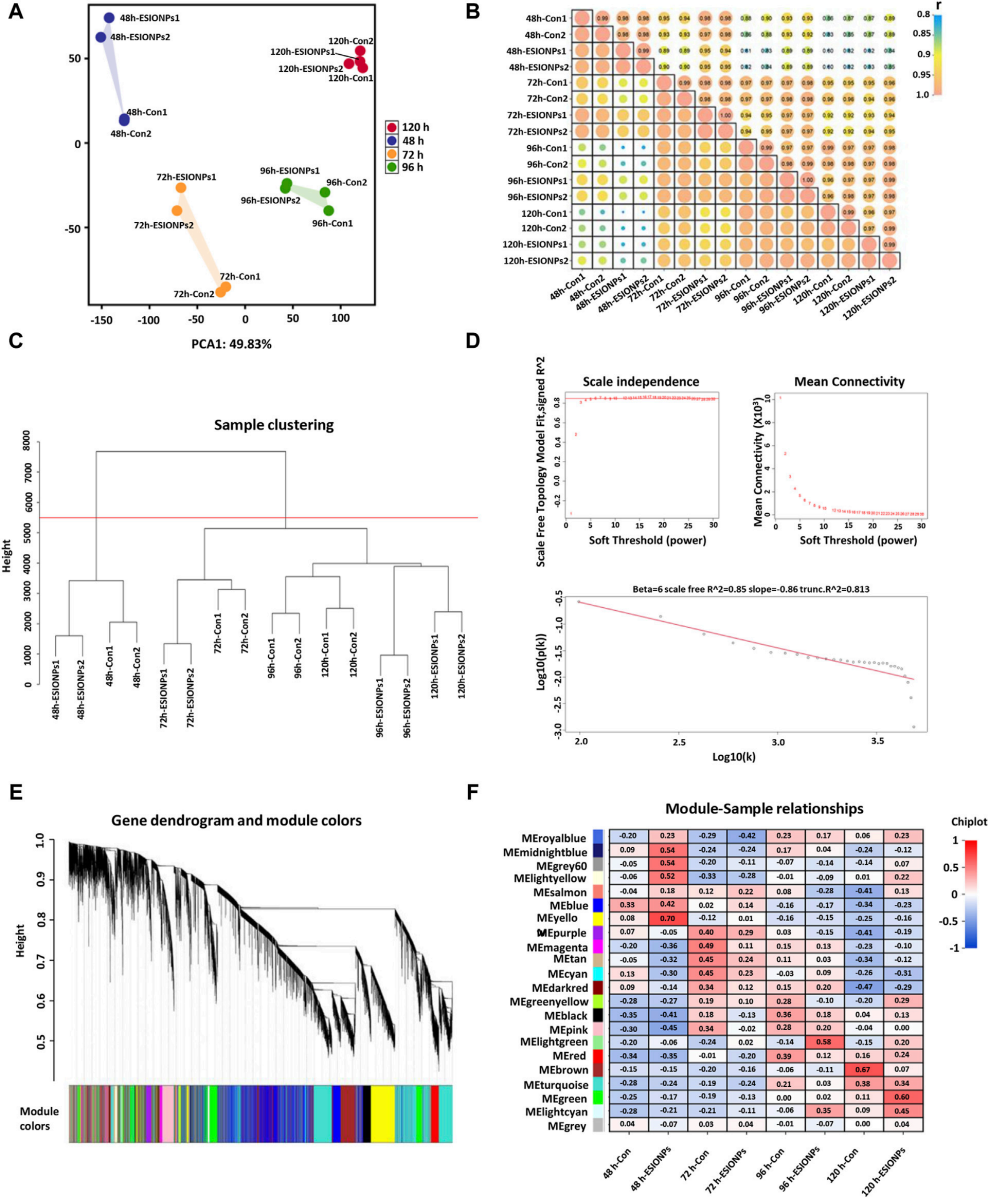
Stage-Specific Gene Co-Expression Networks via WGCNA. **(A)** PCA analysis of all RNA sequencing samples. **(B)** Pearson correlation co-efficient analysis of all RNA sequencing samples. **(C)** Hierarchical clustering information of all RNA sequencing samples. **(D)** The determination of soft threshold power, when β = 6, the scale-free network fitting. **(E)** Based on the hierarchical clustering and adjacency dissimilarity, a gene clustering tree diagram of 22 modules was obtained. **(F)** Module-sample relationship, where the horizontal axis represents the samples and the vertical axis represents the modules. The numbers in each grid represent the correlation between the modules and the samples.

In order to explore the mechanism of toxicity of ESIONPs on zebrafish embryonic development, the DEGs of the control groups and the ESIONPs-exposed groups at 48 hpf, 72 hpf, 96 hpf, 120 hpf were analyzed by RNA-Seq. To determine the relationship between replicates, the samples were clustered using Principal Component Analysis (PCA) and correlation analysis. The PCA showed high repeatability in duplicate samples (Figure 1A), and the Pearson correlation coefficient for each group of replicates also indicated a high repeatability (|r| ≥ 0.8 for all) (Figure 1B). A total of 32,057 transcripts from 16 samples were fused to construct the co-expression network which was constructed by R packages of WGCNA. Firstly, samples were clustered by *hclust* (h > 5,500), no outlier samples were found in the hierarchical clustering (Figure 1C). In order to build a network with scale-free distribution and preserve the information of DEGs as much as possible, we found the best soft-thresholding powers β (β = 6). The connectivity between genes in the network is relatively high (β = 6, *R* ^2^ = 0.813), indicating that the network was scale-free (Figure 1D). Genes were divided into 22 modules (Figure 1E; Supplementary Table S2). In order to study the mechanism of toxicity of ESIONPs at each development stages, the module with the highest correlation of changes was selected at each stage. The yellow module (|*R* ^2^| = 0.62), magenta module (|*R* ^2^| = 0.3765), lightgreen module (|*R* ^2^| = 0.725), and brown module (|*R* ^2^| = 0.6) showed the highest correlation of changes at 48 hpf, 72 hpf, 96 hpf, and 120 hpf, which were used for further research (Figure 1F).

### Functional annotations of key modules to each development stages

Genes in key modules were screened out according to the eigengene-based connectivity (kME) values (|kME| > 0.9). There were 61 hub genes in the yellow module, 128 hub genes in the magenta module, 54 hub genes in the lightgreen module, and 1,157 hub genes in the brown module (Supplementary Table S3).

In the enrichment analysis of the GO pathways, we presented top 10 GO annotations (Supplementary Figures S2A–D; Supplementary Tables S4–S7). The important terms of enrichment in the yellow module (48 hpf) were related to neuron system and muscle development (Supplementary Figure S2A). The terms of related neuron development also mainly were enriched in the magenta module (72 hpf) (Supplementary Figure S2B). The terms of inflammatory were enriched in the lightgreen module (96 hpf, mainly including inflammatory response and chemotaxis of immunocyte (Supplementary Figure S2C). The terms of neuronal signal transmission were enriched in the brown module (120 hpf), including (Supplementary Figure S2D).

In enrichment analysis of KEGG pathways (Supplementary Figures S2E–H; Supplementary Tables S4–S7), yellow module genes (48 hpf) were mainly enriched in embryonic development key signaling pathways, including notch signaling pathway, wnt signaling pathway, hedgehog signaling pathway (Supplementary Figure S2E). The terms of hormone secretion and metabolism were enriched in the magenta module (72 hpf), including pentose phosphate pathway, ubiquitin mediated proteolysis, protein processing in endoplasmic reticulum, GnRH signaling pathway, Endocytosis (Supplementary Figure S2F). Lightgreen module genes (96 hpf) were enriched in metabolism pathways (Biosynthesis of nucleotide sugars, arachidonic acid metabolism, amino sugar and nucleotide sugar metabolism, glycerophospholipid metabolism), necroptosis, C-type lectin receptor signaling pathway, and ferroptosis (Supplementary Figure S2G). Brown module genes (120 hpf) were enriched in pathways related to neuronal signal transmission such as calcium signaling pathway, neuroactive ligand-receptor interaction, cell adhesion molecules, and oxidative phosphorylation (Supplementary Figure S2H).

The enrichment analysis of modules corresponding to each stage of embryonic development by GO and KEGG indicated that ESIONPs might mainly be toxic to the nervous system development, neural conduction, and motor system of zebrafish, and might induce inflammation and ferroptosis in zebrafish embryos.

### Hub genes identified in each module by WGCNA

**FIGURE 2:**
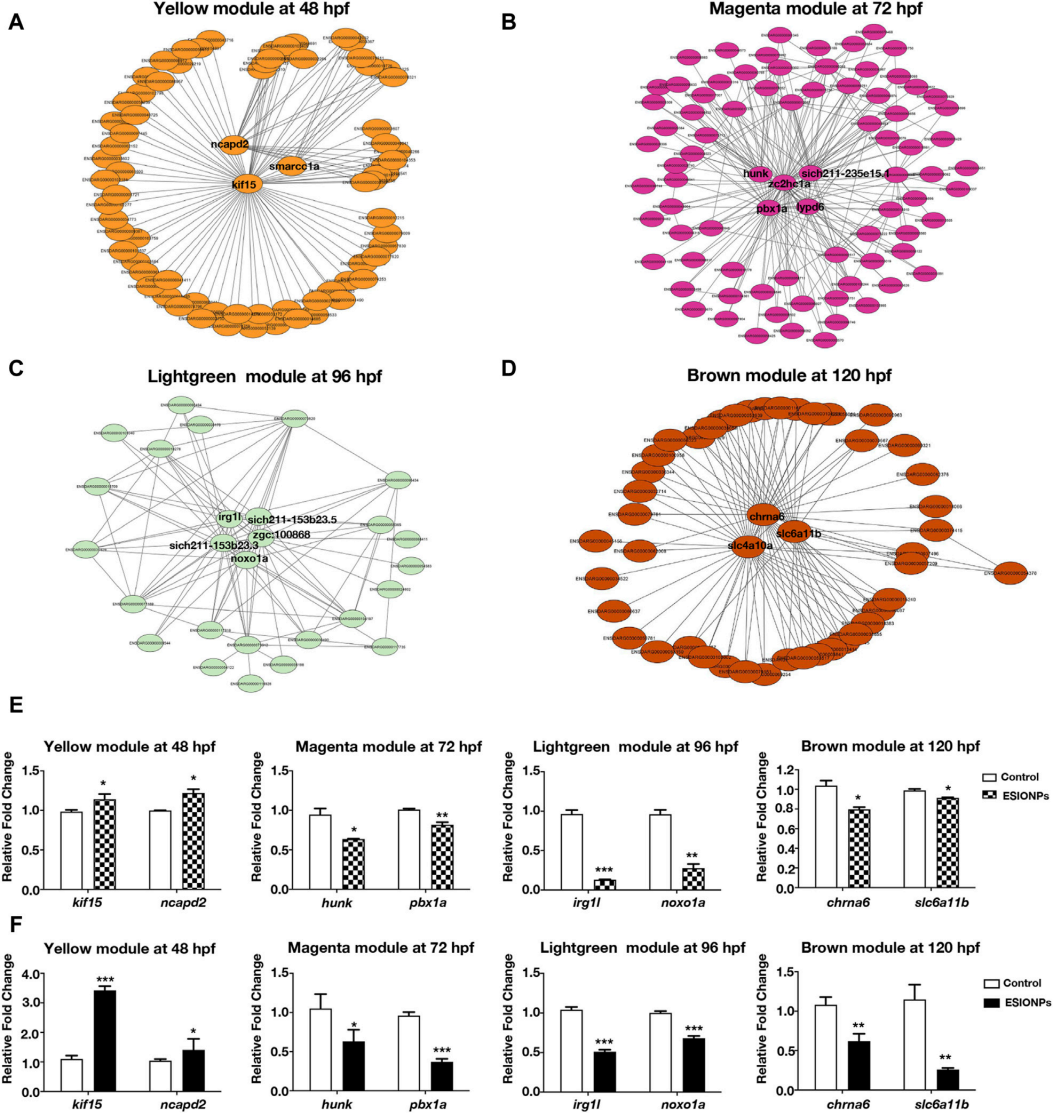
Networks of genes in different modules. **(A)** The key hub genes were shown in Yellow Module (*ncapd2*, *smarcc1a*, *kif15*). **(B)** The key hub genes were shown in Magenta Module (*hunk*, *pbx1a*, *zc2hc1a*, *lypd6*, *sich211-235e15.1*). **(C)** The key hub genes were shown in Lightgreen Module (*irg1l*, *sich211-153b23.5*, *noxo1a*, *sich211-153b23.3*, *zgc:100868*). **(D)** The key hub genes were shown in Magenta Module (*chrna6*, *slc4a10a*, *slc6a11b*). **(E,F)** Gene expression of the key hub genes in 4 modules by RNA-seq **(E)** and qRT-PCR **(F)**. Value are normalized to Control group, and represent mean ± SE from three independent experiments, * *p*-value < 0.05, ** *p*-value < 0.01, *** *p*-value < 0.001 (Student’s *t*-test).

To investigate the mechanism of toxicity of ESIONPs on zebrafish embryos, we next filtered out the hub genes affected by ESIONPs. Networks were constructed to explore relationships among hub genes, which were used as nodes of the scale-free network and had the highest correlation. The top hub genes as the most important nodes in each module were identified and highlighted (Figure 2). There were 3 hub genes in the yellow module: *kif15*, *ncapd2*, *smarcc1a* (Figure 2A), 5 hub genes in the magenta module: *zc2hc1a*, *hunk*, *pbx1a*, *lypd6*, *si:ch211-235e15.1* (Figure 2B), 5 hub genes in the lightgreen module: *irg1l*, *si:ch211-153b23.3*, *si:ch211-153b23.5*, *zgc:100868*, *noxo1a* (Figure 2C), and 3 hub genes in the brown module: *chrna6*, *slc4a10a*, *slc6a11b* (Figure 2D). Although some hub genes (*si:ch211-235e15.1*, *si:ch211-153b23.3*, *si:ch211-153b23.5*, *zgc:100868*) of the magenta and lightgreen modules remain unannotated, the other hub genes of each module could reflect the toxicity of ESIONPs to embryos. For example, genes involved in neuron development (*kif15*, *ncapd2*, *smarcc1a*, *hunk*, *pbx1a*, *lypd6*), neurotransmission (*chrna6*, *slc4a10a*, *slc6a11b*), immune system regulation (*irg1l*), and oxygen stress (*noxo1a*). For each module, two hub genes were selected for validation by qRT-PCR (Figure 2F), which exhibited similar expression trends to the RNA-Seq profiles (Figure 2E). The WGCNA analysis indicated that ESIONPs might have neurotoxicity, which could damage neuron development, nerve conduction, and synaptic transmission. In addition, ESIONPs might also cause ferroptosis in zebrafish embryos.

### ESIONPs resulted in neurotoxicity in zebrafish embryos

**FIGURE 3:**
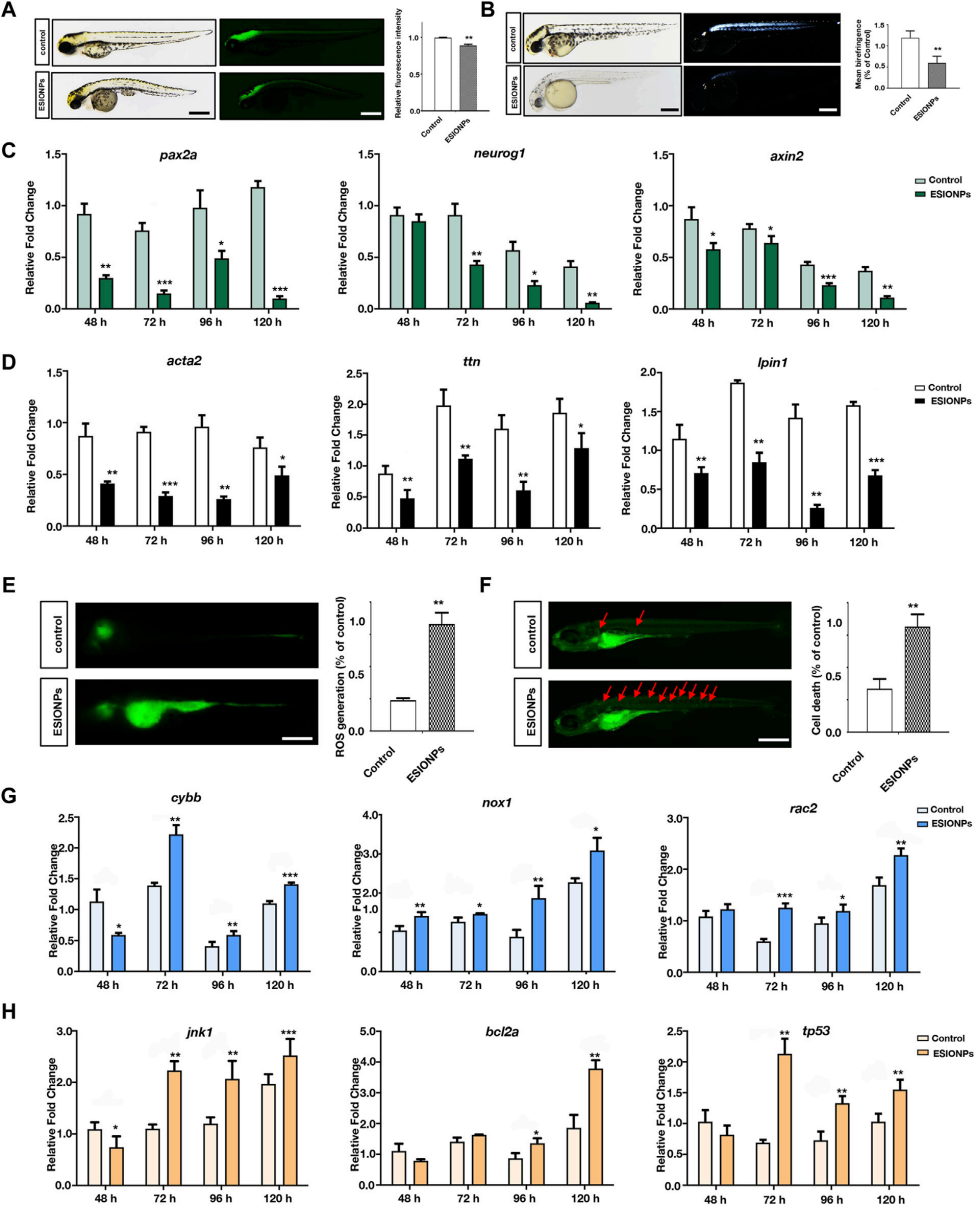
The neurotoxicity and Ferroptosis identified in zebrafish embryos. **(A)** Neuron system fluorescence signals of control group and ESIONPs-exposed group in transgenic zebrafish *Tg* (*eef1a1l1:EGFP*). **(B)** Polarized light intensity of control group and ESIONPs-exposed group of zebrafish muscles. **(C)** The expression of neuron-developmental markers (*pax2a*, *neurog1*, and *axin2*) at different developmental stages of zebrafish. **(D)** The expression of muscle markers (*acta2*, *ttn*, *lpin1*) at different developmental stages of zebrafish. **(E)** ROS generation in zebrafish embryos was detected with fluorescent probe DCFH-DA staining. **(F)** The prevalence of apoptosis in zebrafish embryos was detected with fluorescent dye AO staining; red arrows indicate the apoptotic cells. **(G)** The expression of oxidative stress biomarkers (*cybb*, *nox1*, *rac2*) at different developmental stages of zebrafish. **(H)** The expression of apoptosis biomarkers (*jnk1*, *bcl2a*, *tp53*) at different developmental stages of zebrafish. The fluorescence intensity and polarized light intensity was quantified for individual zebrafish using ImageJ analysis. Scale bar, 200 μm. Value are normalized to Control group, and represent mean ± SE from three independent experiments, * *p*-value < 0.05, ** *p*-value < 0.01, *** *p*-value < 0.001 (Student’s *t*-test).

In *Tg* (*eef1a1l1:EGFP*) transgenic zebrafish embryos, ESIONPs-exposed embryos exhibited significant abnormal development in the nervous system at 72 hpf (Figure 3A), compared with the control embryos. Furthermore, the expression of neuron developmental markers (*pax2a*, *neurog1*, *axin2*) was significantly downregulated in ESIONPs-exposed embryos (Figure 3B). The analysis of the movement track at 120 hpf showed that the movement ability of the ESIONPs-exposed group was significantly weakened (Supplementary Figure S3A), and the expression of the neuromuscular junction and synapse markers (*lrp4*, *musk*, *mpz*) was also downregulated in ESIONPs-exposed embryos (Supplementary Figure S3B). Moreover, the muscle polarization of zebrafish larvae exposed to ESIONPs was significantly reduced (Figure 3C), and the expression of muscle markers (*acta2*, *ttn*, *lpin1*) was also downregulated (Figure 3D). These results showed that ESIONPs not only impaired neuron development, synaptic signal transmission, and neuromuscular junction signal transmission, but also reduced muscle development.

### Ferroptosis induced by ESIONPs could lead to oxidative stress, cell apoptosis and inflammatory response in zebrafish embryos

Next, we determined whether ferroptosis occurred by detecting oxidative stress and apoptosis in zebrafish embryos (Dixon et al., 2012). The oxidative stress was significantly increased in the ESIONPs-exposed embryos (Figure 3E), and the expression of oxidative stress markers (*cybb*, *nox1*, *rac2*) was significantly upregulated in ESIONPs-exposed embryos (Figure 3G). The ESIONPs-exposed embryos also displayed significant cell apoptosis (Figure 3F), and the expression of apoptotic markers (*jnk1*, *bcl2a*, *tp53*) was significantly upregulated (Figure 3H). In addition, the expression of inflammatory markers (*il1b*, *il6*, *tnfa*) was also increased significantly (Supplementary Figure S3C). These results revealed that ESIONPs could induce ferroptosis, resulting in oxidative stress, cell apoptosis, and inflammatory response in zebrafish embryos.

## Discussion

Iron oxide nanoparticles typically consist of a core of magnetic iron oxide surrounded by a stable coating. Fe_3_O_4_, Fe_2_O_3_, and FeO nanoparticles containing iron ions of different valences all belong to iron oxide nanoparticles (Laurent et al., 2008). However, the iron ions released from iron oxide nanoparticles are toxic. The iron ions released from iron oxide nanoparticles and can lead to iron accumulation, oxidative stress and protein aggregation in the neural cells (Yarjanli et al., 2017). It is reported that iron overload may decrease the AChE activity in the brains and livers of zebrafish, which are highly susceptible to iron exposure (Sant Anna et al., 2011). Iron overload zebrafish also exhibit dysregulation in metal homeostasis and decreased neurophysiological performance (Hassan and Kwong, 2020). In this study, after exposure to ESIONPs, the neuron development of zebrafish embryos was significantly disrupted, the motor ability mediated by nerve impulses was also reduced, and ferroptosis might be induced. These phenotypes were similar to organ damage caused by iron ions released from iron oxide nanoparticles. Therefore, we assumed that the neurotoxicity of ESIONPs is mainly caused by the release of iron ions into the environment.

The release and accumulation of ESIONPs in the environment significantly endanger water ecosystems, aquatic organisms, and human health. The small size and high surface activity of NPs allow them to persist in aquatic environments, evade conventional water treatments, and accumulate in aquatic organisms, posing risks to the entire ecosystems (Auffan et al., 2009). ESIONPs can disrupt cellular processes, induce oxidative stress, damage structural integrity, and thus affect the health and reproduction of organisms. In humans, ESIONPs might enter the human body through inhalation or skin contact during water-related activities, arising certain risks to respiratory and other organ systems (Mahmoudi et al., 2011). Our results also showed that ESIONPs had significant neurotoxic effects, which might affect neurological function and lead to degenerative changes or behavioral abnormalities. Therefore, it is imperative that future efforts should focus on reducing the toxicity of ESIONPs while preserving their advanced imaging capabilities to improve their biosafety.

Because of the obvious toxicity of iron oxide nanoparticles, which is caused by the release of iron ions, synthetic and coating strategies have been continuously modified to reduce this toxicity. Iron oxide nanoparticles synthesized by Pouteria caimito fruit can significantly reduce cytotoxicity (Veeramani et al., 2022), and Ag also can reduce the toxicity of Fe3O4 in iron oxide nanoparticles synthesizing (Qi et al., 2022). The neurotoxicity of iron oxide nanoparticles in clinical application also can be reduced by Quercetin in conjugated form as supplementation (Bardestani et al., 2021). Recently, an iron nanoparticle (3 nm in diameter) modified with polyethylene glycol-ethoxy-benzyl ligand on the surface (MnFe_2_O_4_-EOB-PEG) was reported that it can substantially reduce the risk of potential neurotoxicity in rabbits, pigs and macaques (Zhang et al., 2023). Thus, employing synthesis methods with low biological toxicity and various coating techniques for iron oxide nanoparticles might be effective ways to reduce their toxicity.

Transcriptome analysis is the main technology used for toxicity investigation (Teeguarden et al., 2014; Zheng et al., 2018; Arsiwala et al., 2022). However, studies on the toxicity of ESIONPs often focus on a single stage, neglecting the dynamics of embryonic development. The toxicity analysis of a single stage results in many potential or critical factors caused by ESIONPs being concealed. To avoid this problem, this study used WGCNA for toxicity analysis at different stages of embryonic development, which reflected the dynamic impact of ESIONPs on the development of zebrafish embryos. Here, key hub genes in yellow, magenta, lightgreen, and brown modules corresponding to the developmental stages of 48 hpf, 72 hpf, 96 hpf, and 120 hpf were identified. Meanwhile, the expression trends of these hub genes were consistent with the neural development, neural signal transformation, and ferroptosis. Hence, dynamic and continuous analysis of the toxicity of nanoparticles during embryonic development could comprehensively identify key hub genes or novel biomarkers.

Iron is considered an important target for neurodegenerative diseases, and ferroptosis is a type of iron dependent cell death (Yan et al., 2021; Li et al., 2023). An increasing number of studies have confirmed that ferroptosis is associated with the pathological changes of neurological diseases such as Alzheimer’s disease, Parkinson’s disease, and Huntington’s disease, mainly manifesting as neuronal cell death, neuronal loss, and synaptic damage (Li et al., 2022). In this study, ESIONPs caused oxidative stress and cell apoptosis in the neuronal system, ultimately leading to movement disorders, similar to human neurological disorders induced by ferroptosis. It suggested that the use of ESIONPs might induce ferroptosis in the human brain, leading to neuronal damage and death, and increasing the probability of neurological disease occurrence and development.

In this study, through WGCNA analysis, we revealed that exposure to ESIONPs could lead to neurodevelopmental abnormalities and ferroptosis. Since ESIONPs can enter the circulatory system and may have an impact on various body organs. Therefore, a comprehensive evaluation of the safe dosage, *in vivo* distribution, and potential toxicity to other organs is still needed to ensure its safety in clinical applications.

## Scope statement

With the increasing use of iron oxide nanoparticles as contrast agents in clinical practice, extremely small iron oxide nanoparticles (<5 nm in diameter) (ESIONPs) have been synthesizing and modified for better absorption and imaging. However, the toxicity of IONPs might lead to chronic neurological and motor system diseases, so research on the toxicity of ESIONPs is urgent. Here, we used zebrafish as a model animal to explore the potential toxicity of ESIONPs on embryonic development. By performing RNA-Seq on control and ESIONPs-exposed embryos at 48 hpf, 72 hpf, 96 hpf, and 120 hpf, WGCNA analysis revealed different module corresponding to each embryonic development stage, and key biomarkers were identified in each module. The expression trends of these key biomarkers were further validated by qRT-PCR. Moreover, exposure to ESIONPs might disrupt the neuronal and muscle development of zebrafish, and induced ferroptosis, leading to oxidative stress, cell apoptosis, and inflammatory response in zebrafish larvae. The toxicity study of ESIONPs herein provides certain suggestions for the potential clinical application of ESIONPs.

## Data Availability

The raw sequence data reported in this paper have been deposited in the Genome Sequence Archive in National Genomics Data Center, China National Center for Bioinformation/Beijing Institute of Genomics, Chinese Academy of Sciences (GSA: CRA016266) that are publicly accessible at https://ngdc.cncb.ac.cn/gsa.
