# Supplementary figures and images for: Comparative transcriptomics revealed neurodevelopmental impairments and ferroptosis induced by extremely small iron oxide nanoparticles
Source: Front Genet. 2024 May 17;15:1402771. doi: 10.3389/fgene.2024.1402771 (PMC11140123; doi:10.3389/fgene.2024.1402771)

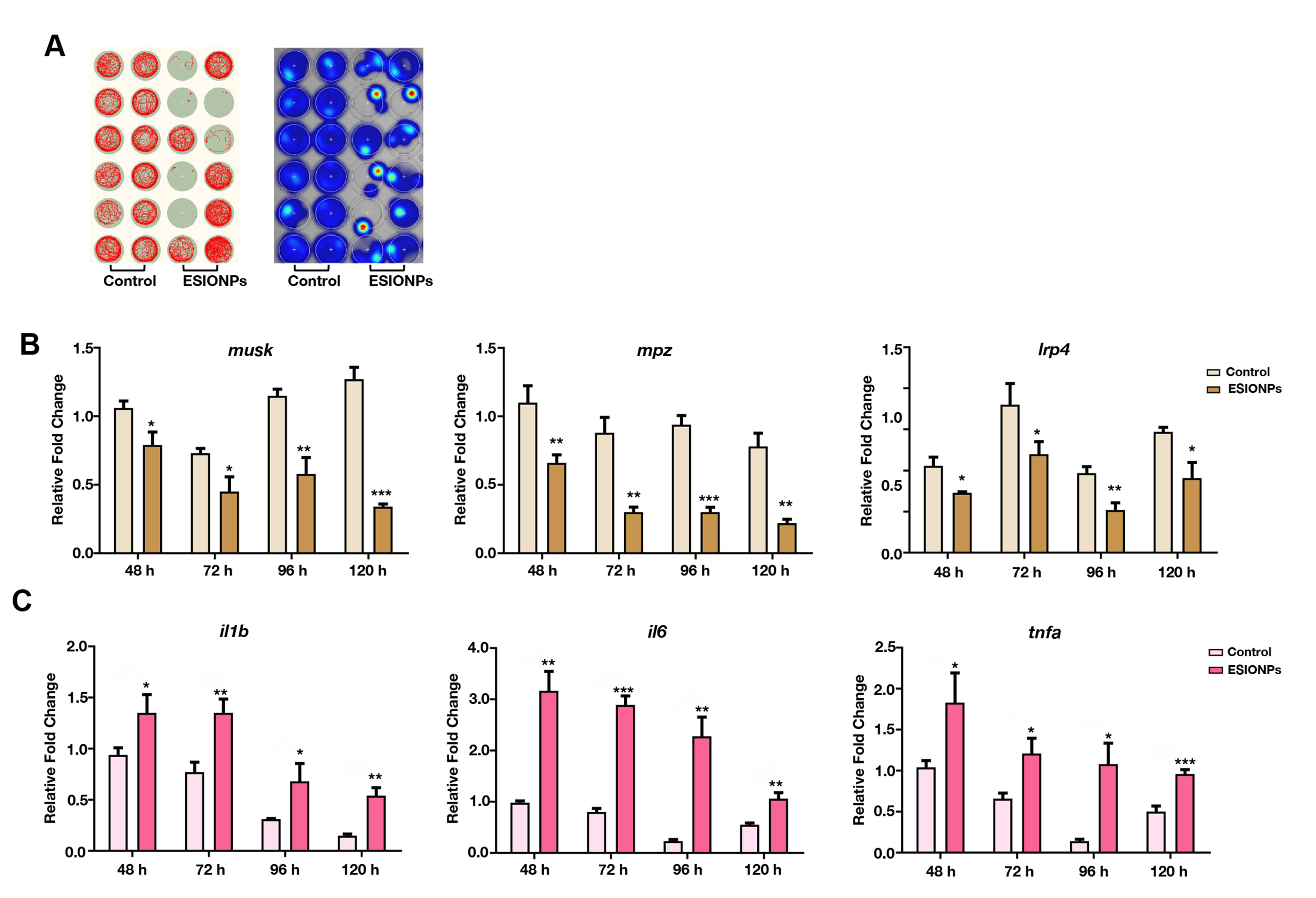

Supplement: Supplementary file 1 [file Image3.TIF]

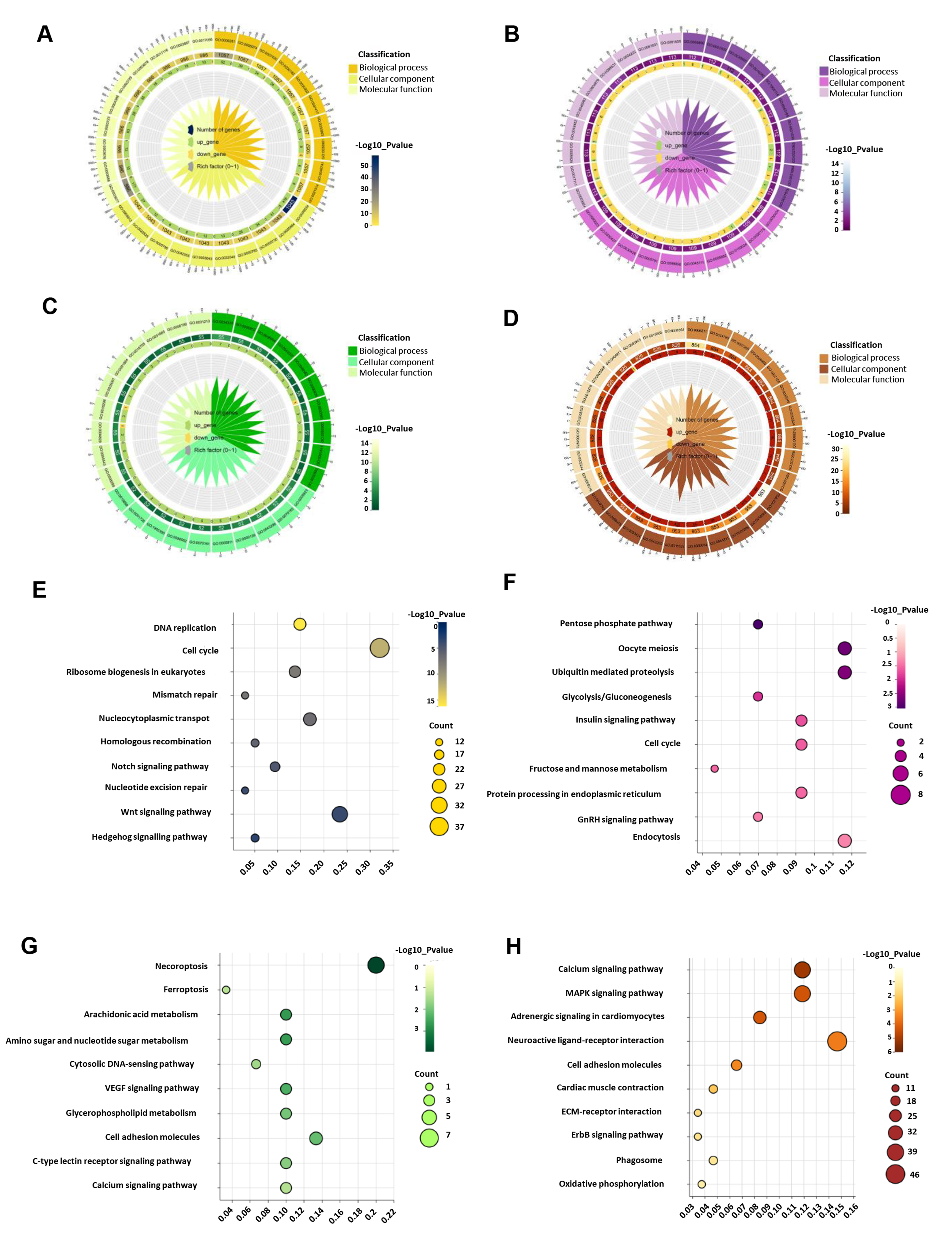

Supplement: Supplementary file 2 [file Image2.TIF]

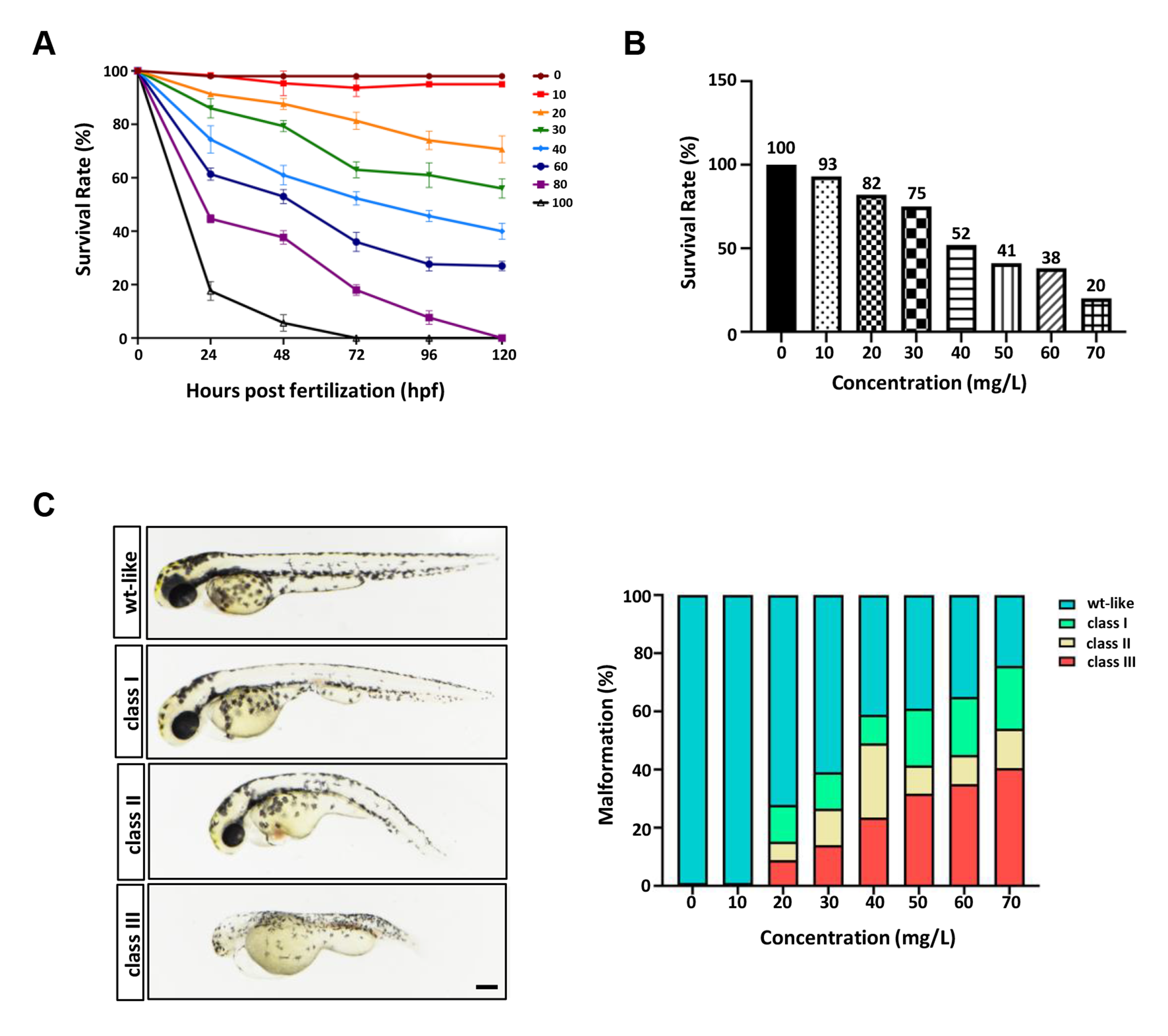

Supplement: Supplementary file 3 [file Image1.TIF]
